# Vaccination with toxofilin DNA in combination with an alum-monophosphoryl lipid A mixed adjuvant induces significant protective immunity against *Toxoplasma gondii*

**DOI:** 10.1186/s12879-016-2147-1

**Published:** 2017-01-05

**Authors:** Pengxia Song, Shenyi He, Aihua Zhou, Gang Lv, Jingjing Guo, Jian Zhou, Yali Han, Huaiyu Zhou, Zhen Hao, Hua Cong

**Affiliations:** 1 Department of Parasitology, Shandong University School of Medicine, Jinan, Shandong Province 250012 People’s Republic of China; 2 Shandong University School of Medicine, 250021 Jinan, Shandong Province People’s Republic of China; 3 Department of Pediatrics, Provincial Hospital Affiliated to Shandong University, Shandong University School of Medicine, 250021 Jinan, Shandong Province People’s Republic of China

**Keywords:** *Toxoplasma gondii*, Toxofilin, Bioinformatics, DNA vaccine, Adjuvant

## Abstract

**Background:**

A widely prevalent disease, toxoplasmosis poses serious health threats to both humans and animals; therefore, development of an ideal DNA vaccine against *Toxoplasma gondii* is needed eagerly. The purpose of the present study is to assess the protective efficacy of a DNA vaccine encoding the *T. gondii* toxofilin gene (pEGFP-toxofilin). In addition, toxofilin DNA vaccine combined with the individual adjuvants, alum or monophosphoryl lipid A (MPLA), or a mixture of alum-MPLA adjuvant were screened for their ability to enhance antibody responses.

**Methods:**

Using bioinformatics, we analyzed the gene and amino acid sequences of the toxofilin protein, recognizing and identifying several potential linear B and T helper (Th)-1 cell epitopes. BALB/c mice were immunized three times with either toxofilin DNA vaccine alone or in combination with the adjuvants such as alum, MPLA or an alum-MPLA mixture. The systemic immune response was evaluated by cytokine, the percentage of CD4 (+) and CD8 (+) T cells and specific antibody measurement. Two weeks after the last immunization, protective efficacy was evaluated by challenging intraperitoneally with 1 × 10^4^ tachyzoites of *T. gondii* or intragastrically with 20 cysts of *T. gondii* PRU strain.

**Results:**

All experimentally immunized mice developed strong humoral and cellular immune responses compared with the control groups. Moreover, by comparison with the non-adjuvant toxofilin DNA vaccine group, adding alum adjuvant to toxofilin DNA vaccine resulted in an increase in humoral response and a skewed Th2 response. However, the MPLA adjuvant with toxofilin DNA vaccine induced significantly enhanced humoral and Th1-biased immune responses. Importantly, the co-administration of alum-MPLA adjuvant in combination with the toxofilin DNA vaccine shifted the Th2 immune response to a Th1 response compared with the alum-toxofilin group, and elicited the strongest humoral and Th1 responses among all the groups. At the same time, a longer survival time and less cyst amounts against *T. gondii* infection were also observed in the alum-MPLA-toxofilin group in comparison with single or no adjuvant groups.

**Conclusions:**

*Toxoplasma gondii* toxofilin is a promising vaccine candidate that warrants further development. Co-administration of the alum-MPLA adjuvant mixture with DNA vaccine could effectively enhance immunogenicity and strongly skew the cellular immune response towards a Th1 phenotype.

## Background

*Toxoplasma gondii* is an obligate intracellular protozoan parasite [1]. It has a complex life cycle, infecting a broad host range of mammals and birds as intermediate hosts but with felids as the only definitive hosts [2]. The T.*gondii* has a wide range of distribution and high infection rates in many parts of the world and a third of population is infected [3, 4]. Although infection with *T. gondii* may be asymptomatic in immunocompetent individuals, severe disease can develop in immunocompromised patients or fetuses of seronegative women undergoing primary infection during pregnancy [4, 5].

The primary strategy for the treatment of toxoplasmosis is chemical drugs; however, such drugs have many drawbacks, including poor tolerance, side effects, and the development of drug resistance and are ineffective for the treatment of *T. gondii* tissue cysts in warm-blooded animals. Besides, they can’t prevent reinfection [6]. Therefore, the development of an effective and safe vaccine against toxoplasmosis is of great importance to prevent the continued spread of this disease. In the past 15 years, significant progresses have been made in the development of vaccine [7]. DNA vaccinations against toxoplasmosis are well known to induce Th1 immune response [8]. Several single-antigen vaccines such as ROP8, TgCyP, and GRA6, have been successful in facilitating survival following a lethal dose of infection challenge, with a range of survival rates from 37.5 to 50.0% [9–11]. However, the development of a higher efficacy vaccine remains a vital challenge in light of the persistent nature of chronic toxoplasmosis.

Toxofilin, which was discovered in the rhoptry proteome, was originally based on its role in binding and the regulation of host actin dynamics. Recently, toxofilin was crystallized in complex with mammalian actin, establishing the interaction between the parasite and host protein [12]. Toxofilin is an internal protein. During the parasites invade into the host cell, the protein was secreted to host cell to facilitate vacuole folding and tachytozoites invasion. In addition, anti-toxofilin or anti-HA antibodies was detected in infected host cells by fluorescence microscopy imaging [13]. However, there is thus far no evidence as to whether toxofilin DNA vaccine can induce significant humoral and cellular responses in mice and the protective efficacy of toxofilin DNA as a vaccine against *T. gondii* remains unclear.

A good vaccine also depends on the choice of adjuvants which play a key factor in dictating the effectiveness of the immune response [14, 15]. Aluminum salts (alum) are the most widely used of adjuvants in vaccine development. However, alum which is used as an adjuvant has several limitations. Obviously, alum-based adjuvants tend to T helper (Th) 2 immunity and are incompatible with small peptide antigens [16]. With the aim of developing a transmission-blocking *T.gondii* vaccine, the application of compound adjuvant has leaded the vaccine development to a new era, resulting in a stronger immune response through a synergistic effect. This strategy incorporating a combination of adjuvants (monophosphoryl lipid A (MPLA) and alum) is proven to be effective [17, 18]. MPLA as an adjuvant system have additive effects, especially intracellular processing of Th1 antigens by the major histocompatibility complex. At the same time, MPLA can enhance antigen-specific induction of antibodies to a variety of antigens [19, 20].

To our knowledge, no report has described a toxofilin-based vaccination regimen against *T. gondii.* In this study, the linear B- and Th-cell epitopes of toxofilin protein were analyzed using bioinformatics. Furthermore, through analysis of the generated peptide segment tables, we constructed a eukaryotic plasmid pEGFP-toxofilin and investigated the overall survival, cysts burden and levels of humoral and cellular immune responses elicited by toxofilin DNA vaccine alone or in combination with one and more adjuvants following intraperitoneal (i.p.) or intragastrically challenge or in BALB/c mice.

## Methods

### Bioinformatic analysis

The gene and amino acid sequences of toxofilin were identified from GenBank (http://www.ncbi.nlm.nih.gov/genbank), the primary characteristics of the gene encoding protein were analyzed by ProtParam (http://web.expasy.org/protparam/), the primary amino acid sequences of toxofilin homologs were downloaded from the National Center for Biotechnology Information, and the multiple sequence alignment was generated using the Clustal W method in DNASTAR MegAlign software.

### Bioinformatic tools for predicting potential B-cell-binding epitopes

B-cell epitopes are recognized in their native structure by B-cell receptors or antibodies. Continuous B cell epitope prediction is mainly according to the amino acid properties. However the discontinuous B cell epitope prediction requires 3D structure of the antigen [21–23]. In this study, the complete amino acid sequence and the secondary structure of toxofilin were analyzed using DNASTAR [24]. A higher score of toxofilin corresponded to a higher probability of the residue being involved in the epitope, using DNAMA software. The SOPMA and I-TASSER online services were used to construct the three-dimensional (3D) structures of the toxofilin epitopes [25].

### Identification of the Th-cell epitopes

The Immune Epitope Database online service (http://tools.immuneepitope.org/analyze/html/mhc_II_binding.html) was used to predict MHC II molecules of toxofilin. It is important to note, however, that such binding to MHC is necessary but not sufficient for recognition by T cells [26].

### Cell culture

HEK239T cells stored at −80 °C were thawed for 1 min at 37 °C followed by culture in Dulbecco’s modified Eagle’s medium (DMEM; GIBCO) with 10% fetal bovine serum, 100 mg/mL streptomycin, and 100 IU/mL penicillin at 37 °C in 5% CO_2_.

Macrophages were cultured at 37 °C in DMEM with 4 mM l-glutamine (Dutscher) supplemented with 10% heat-inactivated fetal calf serum (Dutscher) and Hepes 10 mM (Dutscher), in a 5% CO_2_ atmosphere.

### Mice and parasites

Six- to eight-week-old female BALB/c mice were purchased from Shandong University Laboratory Animal Centre (Jinan, China). All animal experiments were approved with the Animal Ethics Committee of Shandong University under Contract 2011–0015.

We used two strains of *T. gondii* (RH and PRU) in this study. The low virulent strain (PRU strain) of *T. gondii* was kept in our laboratory by passage of cysts in Kunming mice. The tachyzoites of the RH strain of *T. gondii*, which was preserved at −80 °C, were obtained by passage on macrophages. About 1 × 10^4^ tachyzoites of intermediate virulence were collected from one 225-cm^2^ culture flask, corresponding to 1 μg of TE. Tachyzoites of highly virulent *T. gondii* were collected to challenge mice.

### The construction of the expression plasmids

The *T. gondii* toxofilin gene coding sequence [GenBank: AJ132777.2] was amplified by PCR from genomic parasite DNA using the following specific primer pair. Toxofilin for eukaryotic expression plasmid construction: forward primer: 5′-CGGGGTACCATGGCGCAATACAAGTCACG-3’(*Kpn* I), reverse primer: 5’-CG GGATCCTTACGACGAGGGCATAGCG-3’(*BamH* I). Toxofilin for prokaryotic expr -ession plasmid construction: forward primer: 5′-CGGGGTACCATGGCGCAA TACAAGTCACG-3’(*Kpn* I), reverse primer: 5′-CGGCGGCCGCTTACGACGA GGGCATAGCG-3’′ (*Not* I) (recognition sites for *Kpn* I, *BamH* I and *Not* I, respectively, are underlined). The amplified PCR products were cloned into the eukaryotic expression vector pEGFP-C1 (TransGen, China) and the prokaryotic expression plasmid pET-30a(+) to formed a recombinant plasmid, pEGFP-toxofilin and pET30-toxofilin.

### pEGFP-toxofilin plasmids expression in vitro

*p*EGFP-toxofilin plasmids were transformed into *Escherichia coli* DH5 by the way of hot shot (42 °C.). Expression of two genes in transformed DH5α cells was screened on LK solid medium, followed by choosing a single colony to cultivate for harvest of recombinant plasmids. All the recombinant plasmids were extracted by an endotoxin-free plasmid purification kit (Tiangen, China) and stored at −20 °C until use. The plasmids concentrations were determined by A260/A280 measurement.

HEK239T cells grown in 6-well plates were respectively transfected with recombinant eukaryotic plasmid (pEGFP-toxofilin) or an empty plasmid pEGFP-C1 with the assistance of LipofectamineTM 2000 reagent (Invitrogen, USA) in accordance with the manufacturer’s instructions. Protein expression in vitro was observed by specific green fluorescence on HEK293T cells after incubation for 24 h at 37 °C in a 5% CO_2_ incubator. At 36 h post-transfection, the cells were rinsed twice with PBS, handled with lysis buffer (Sigma, USA), and centrifuged at 16,000 × g for 30 min. The lysates of transfected cells were analyzed by western blotting.

### Expression and purification of pET30- toxofilin in bacteria

*Escherichia coli* DH5α strain cells were transformed by recombinant pET30-toxofilin and grown in Luria Bertani (LB) at 37 °C until the cell density reached an absorbance of 0.6 (at λ = 600 nm). Synthesis of recombinant pET30-toxofilin protein was induced with 1 mM isopropyl-β-d-thiogalactoside (IPTG) for 6 h and 8 h at 25 °C and the growth for the next 3 h at 37 °C. The cells were lysed and the protein was purified. The endotoxin was removed with the ToxinEraser™ Endotoxin Removal Kit and measured by the Chromogenic End-point Endotoxin Assay Kit (Chinese Horseshoe Crab Reagent Manufactory, Xiamen, China). Expression of the pET30-toxofilin protein was analyzed by western blotting and kept at −80 °C for further use.

### Immunization and challenge

The immunization experiments that pEGFP-toxofilin DNA was used as the vaccine were performed in 6- to 8-week-old female BALB/c mice, which were randomly divided into eight groups (n = 25/group). Mice were injected intramuscularly three times at fortnightly intervals with the following four experimental immunizations: non-adjuvanted toxofilin (50 μg toxofilin DNA + 100 μL PBS/mouse); alum-toxofilin (50 μg toxofilin DNA + 50 μg alum + 50 μL PBS/mouse); MPLA-toxofilin (50 μg toxofilin DNA + 50 μg MPLA + 50 μL PBS/mouse); alum-MPLA-toxofilin (50 μg toxofilin DNA + 50 μg MPLA + 50 μL PBS/mouse); and 4 control groups, with mice receiving 150 μL PBS, 150 μg alum, 150 μg MPLA, 150 μg alum-MPLA. The blood of mice in each group was collected at 0, 2, 4, and 6 weeks by orbital blood and sera were stored at −20 °C until used for subsequent analysis. Two weeks after the final inoculation, 12 mice in each group were challenged i.p. with 1 × 10^4^ *T. gondii* RH strain tachyzoites, and the remaining mice in all groups were inoculated with 20 cysts of the attenuated virulent PRU strain orally. The individual survival time injected with RH strain along with the overall percentage survival were recorded. The dead mice infected RH strain were treated by high pressure sterilization and sent to the animal experiment center for further processing. The brain cysts were determined one month later after the challenge infection with 20 cysts of the attenuated virulent PRU strain orally for a one time. Each brain was homogenized in 2 ml PBS, and the mean number of cysts per brain was calculated. This analysis was performed in three independent experiments.

### Determination of antibodies by ELISA

Anti-T. gondii IgG, IgG1, and IgG2a antibodies were detected using enzyme-linked immunosorbent assay (ELISA) in accordance with the manufacturer’s instructions (Southern Biotech Co., Ltd, Birmingham, USA). Briefly, microtiter 96-well plates were coated overnight at 4 °C with pET30-toxofilin protein antigen (diluted in 0.1 M carbonate buffer (pH 9.5) for optimal binding. The plates were washed three times with PBS containing 0.05% Tween20 (PBST) and then blocked with 1% bovine serum albumin for 1 h at 37 °C with gentle shaking. After three washes with PBST, the plates were incubated with mouse sera diluted 1:100 in PBS for 1 h, followed by incubation with goat anti-mouse IgG, IgG1, or IgG2a secondary antibodies for 1 h to detect the target antibody and isotype control, respectively. Finally, the immune enzymatic reaction complexes were visualized by incubating with ortho-phenylenediamine (Sigma, USA) and 0.15% H_2_O_2_ for 30 min. Reactions were stopped by adding 2 M H_2_SO_4,_ and the results were read at OD450 nm using an ELISA plate reader. An average of three independent ELISAs for each serum sample was recorded.

### Cytokine assays

Cytokine levels were determined as previously described [16]. Three mice per group were euthanized and their splenocytes aseptically harvested after two weeks after the last immunization, Cells were dispensed into 96-well plates at a density of 5 × 10^5^ cells at 37 °C with 95% relative humidity in 5% CO_2_. Cell-free supernatants were harvested and assayed for interleukin (IL)-4, IL-10, and IFN-γ at 24 h, 72 h, and 96 h, respectively, using a commercial ELISA kit. All measurements were run in triplicate.

### Identification of CD4+ and CD8+ T cells by flow cytometry

By flow cytomety analysis, the percentage of CD4+ and CD8+ T cell in spleen were determined. The cell concentration was adjusted to 1 × 10^6^ cells/ml in PBS containing 2% FBS. After incubation with FITC-conjugated anti-mouse CD4+ monoclonal antibody (mAb), PE-conjugated anti-mouse CD3+ mAb and Cy5.5-conjugated anti-mouse CD8+ mAb (eBioscience) at 4 °C for 30 min in the dark. The cultures were washed by 2 mL PBS and the suspensions were analyzed using SYSTEM II software (Coulter) through FACScan flow cytometer (BD Biosciences, San Jose, CA, USA).

### Statistical analyses

All statistical analyses and graph plotting were performed by SPSS13.0 Data Editor and the differences in the data (antibody responses, lymphoproliferation assays, cytokine production and brain cyst burden) between all the groups were compared by one-way analysis of variance (ANOVA). Tukey’s studentized range test was used for post-test comparisons. Survival rates for the experimental and control mice were compared using the Kaplan–Meier method and compared with the log-rank test. The differences were considered significantly if *P* < 0.05.

## Results

### Bioinformatic analysis

**Fig. 1:**
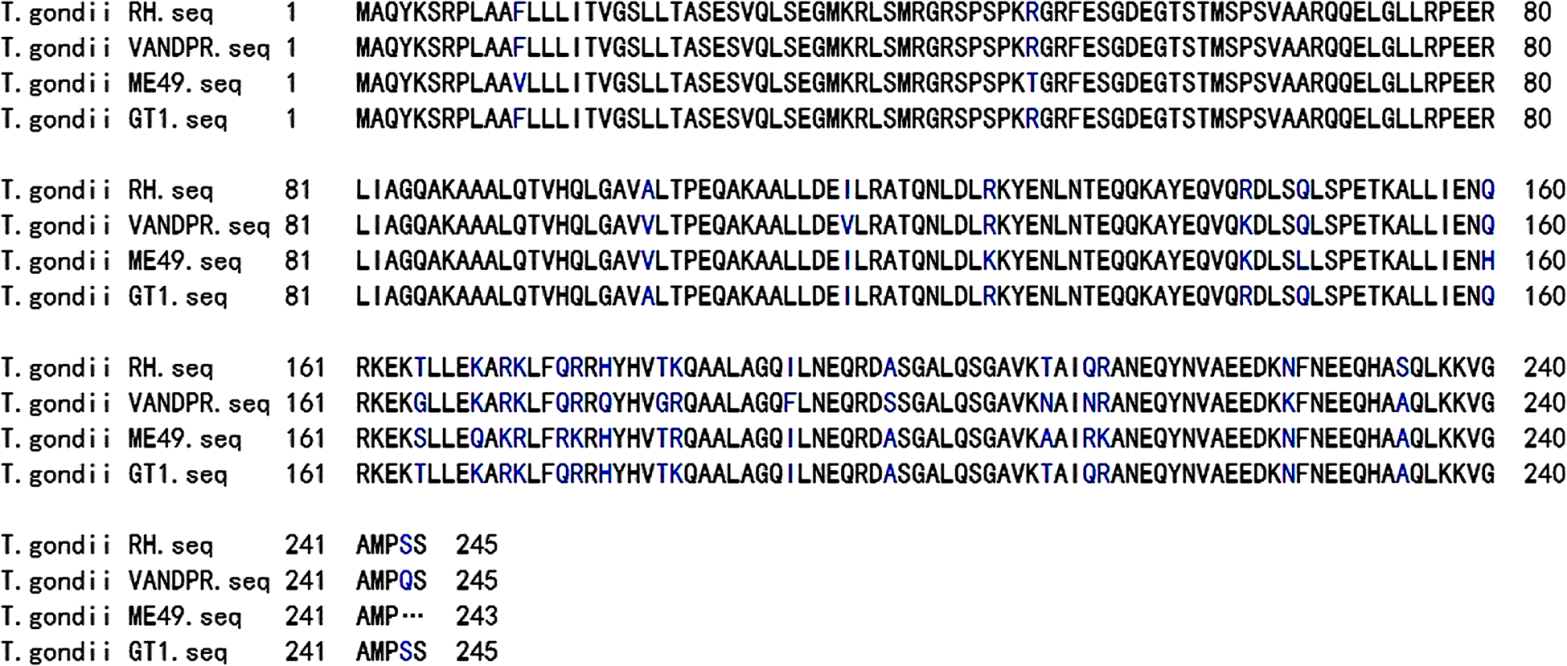
Alignment of toxofilin protein sequences from toxoplasma strains. Blues letters indicate amino acid differences, while … represents a missing amino acid

The toxofilin protein of *T. gondii* (Tg-toxofilin) is a 245 amino acid protein with a molecular weight of 27.1317 kDa. Its physical and chemical properties include a theoretical PI of 9.57, an instability index of 60.41, an aliphatic index of 84.98, and a grand average of hydropathicity of −0.63. For four different *T. gondii* strains, the protein sequence alignment is shown in Fig. 1. and share 96.53% similarity, with toxofilin of the *T. gondii* RH strain sharing 100%, 91.84%, and 94.29% sequence identity with GT1, ME49, and VANDPR strains, respectively.

### Secondary structure and 3D modeling

**Fig. 2:**
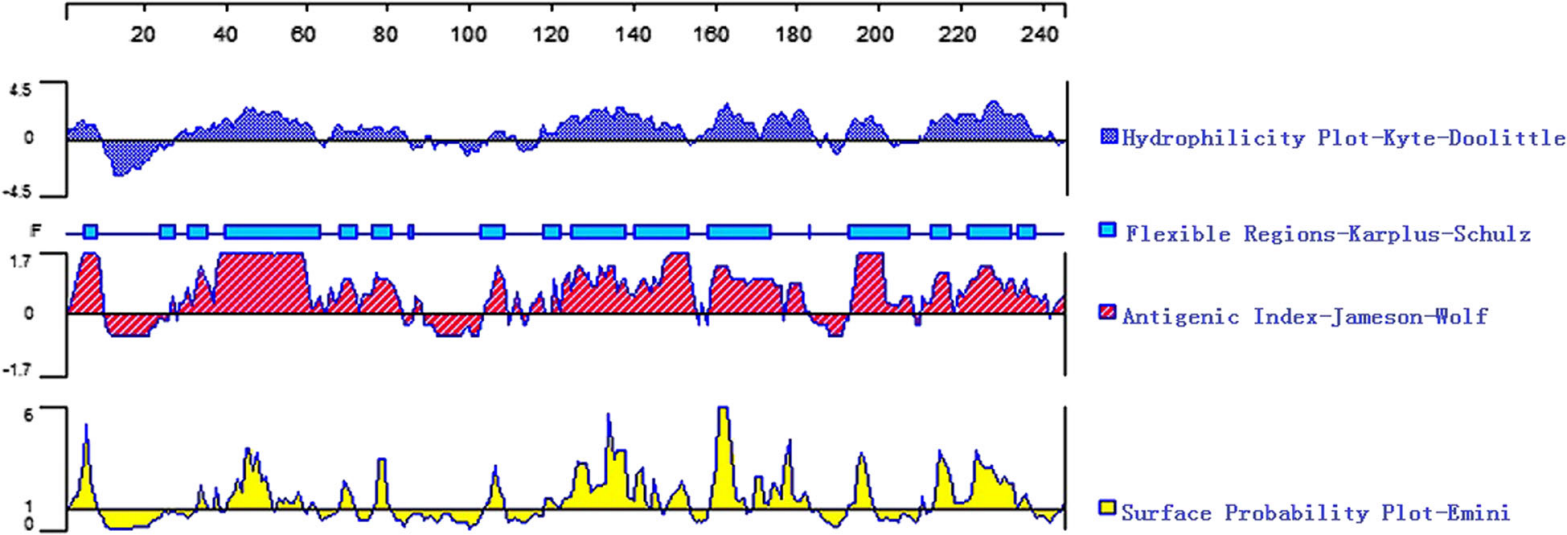
Prediction of B-cell epitopes in the toxofilin protein based on DNAStar. The linear-B cell epitopes of toxofilin predicted by DNASTAR in flexibility, hyd- rophilicity, surface probability and antigenicity index rules. The prediction was run for three times

B-cell epitopes are the portions of antigens that are recognized by the variable regions of antibodies. Accurately predicting epitopes is of great help for designing immunogenic peptides and new vaccines. In the present study, the DNASTAR software was used to predict antigenic index and surface probability, as shown in Fig. 2. Based on this analysis, the peptides with good hydrophilicity, high accessibility, high flexibility, and strong antigenicity were chosen as antigen epitopes. To further confirm these results, the DNAMAN software was used to analyze these sequences and nine potential epitopes were chosen with the highest antigen index scores, as shown in Table 1.

**Table 1:** Analysis of linear-B cell antigenic epitopes on Tg-toxofilin^a^

Order	Position	Sequence	Score^b^
1	5-31	KSRPLAAFLLLITVGSLLTASESVQLS	1.184
2	82-105	IAGQAKAAALQTVHQLGAVALTPE	1.153
3	107-115	AKAALLDEL	1.089
4	175-190	QRRHYHVTKQAALAGQ	1.096
5	199-211	SGALQSGAVKTAI	1.095
6	233-242	SGALQSGAVKTAI	1.095
7	71-78	ELGLLRPT	1.072
8	62-69	SPSVAARQ	1.068
9	137-158	AYEQVQRDLSQLSPETKALLLE	1.067

^a^The prediction was run for three times. Two or more amino acids condense into a peptide

^b^High score = high binding

**Fig. 3:**
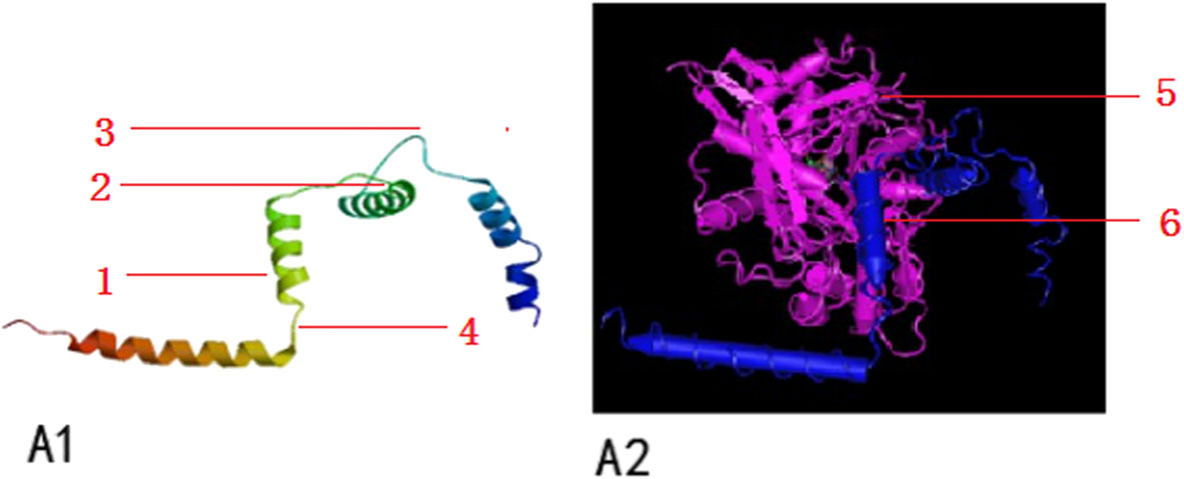
Toxofilin 3D structure predictions. (**A1**) 3D structure of Tg-toxofilin 3D model with the highest score for the Tg-toxofilin protein was selected (1: Alpha helix, 2: Random Coil, 3: Extend Strand, 4: Beta Turn). (**A2**) Complex of Mammalian Actin With Toxofilin From *Toxoplasma gondii*, Toxofilin from toxoplasma gondii forms a ternary complex with an antiparallel actin dimer. The model was viewed by VMD software and colored to illustrate the different protein components (5: Actin, Alpha skeletal Muscle, 6: toxofilin)

A potentially antigenic region of a protein increases the epitope prediction and promote a better understanding the 3D structure, contributing to the elucidation of the relationship between structure and function. The SOPMA and I-TASSER online services were used to predict the 3D structures of toxofilin alone and in complex with mammalian actin (Fig. 3 A1 and A2).

### Identification of the Th-cell epitopes

The Th-cell epitopes identified on toxofilin by bioinformatic analyses reportedly have the ability to bind strongly to MHC class II molecules (Table 2). It is known that the binding force increases can influence Th-cell differentiation and promote more cells to differentiate into Th-1 cells [15]. Therefore, we speculate that toxofilin has the potential to induce Th-1 cell-mediated immune responses.

**Table 2:** Ligation strength analysis of Tg-toxofilin for MHC class II molecules using SYFPEITHI^a^

MHC II Allele^b^	Start-Stop^c^	Sequence	Percent: e Rank^d^
HLA-DRB1*01:01	91-105	LQTVHQLGAVALTPE	0.39
HLA-DRB1*01:01	9-23	LAAFLLLIVGSLLT	0.42
H2-IAb	78-92	EERLIAGQAKAAALQ	3.04
H2-IAb	96-110	QLGAVALTPEQAKAA	6.58
H2-IAd	178-192	HYHVTKQAALAGQIL	0.13
H2-IAd	231-245	QHASQLKKVGAMPSS	0.62
H2-IEd	169-183	KARKLFQRRHYHVTK	13.80
H2-IEd	28-42	VQLSEGMKRLSMRGR	21.98

^a^The prediction was run for three times

^b^H2-IAb, H2-IAd and H2-IEd alleles are mouse MHC class II molecules; the HLADRB1*01:01 allele is a human MHC class II molecule

^c^We chose 15 amino acids for analysis each time

^d^Low percentile = high level binding, high percentile = low level binding

### Identification of restructuring eukaryotic, prokaryotic and pro expression plasmids

**Fig. 4:**
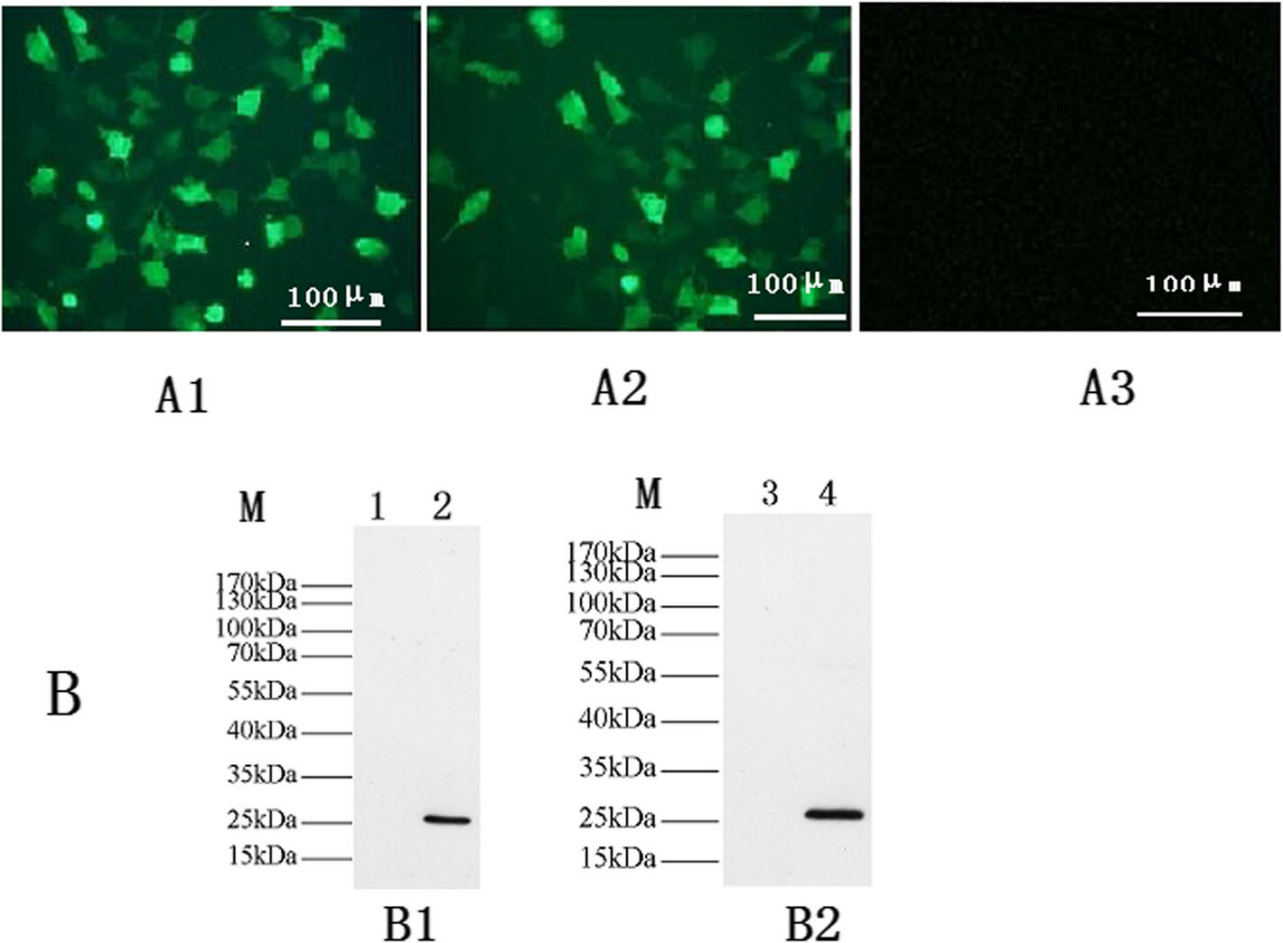
Green fluorescent microscopy image of HEK293T cells and western blotting. (**A1**) HEK293 cells transfected with pEGFP-Tgtoxofilin under blue light. (**A2**) HEK293 cells transfected with pEGFP. (**A3**) non-transfected HEK293 cells. (**B**) Western blot analysis of pEGFP-toxofilin protein in transfected HEK293 cells, PET-30 toxofilin protein in bacterial, respectively. (**B1**) In HEK 293 cells, the expressed proteins were reacted with a *T. gondii* antibody (lanes 2) and the control empty pEGFP plasmid-transfected cells did not show any band with a *T. gondii* antibody (lane 1). (**B2**) PET-30 toxofilin protein were reacted with a *T. gondii* antibody (lane 4), the empty pET30a vector showed no specific bands with a *T. gondii* antibody (lane 3). (lane M) Respresents protein molecular weight markers

Forty-eight hours after transfection of HEK cells, the presence of pEGFP-toxofilin or pEGFP-C1 was determined by fluorescence microscopy. As shown in Fig. 4(A1) and (A2), green fluorescence was observed in HEK293T cells transfected with pEGFP-toxofilin and pEGFP-C1, whereas there was no signal in the untransfected cells Fig. 4 (A3). Western blot analysis of these proteins was shown in Fig. 4B; a specific protein band (about 27 kDa) was recognized in cells transfected with pEGFP-toxofilin by incubation with a *T. gondii* antibody (lanes 2), whereas the negative controls transfected with empty pEGFP vector showed no specific bands upon incubation with a *T. gondii* antibodies (lane 1). The purified recombinant pET30-toxofilin vector was used to produce toxofilin protein antigen. Western blot technique by using a T. gondii antibody was performed to verify the toxofilin antigen (lane4).

### Determination of antibody responses in immunized BALB/c mice

**Fig. 5:**
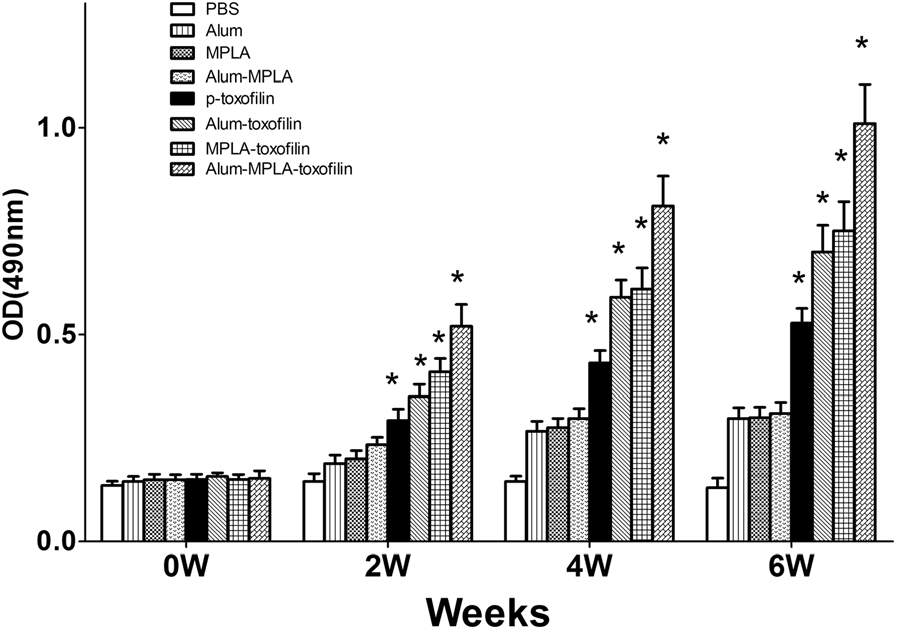
Measurement of specific IgG antibodies in sera of immunized mice. Sera were collected one day prior to each immunization and determined by ELISA. Results are shown as means of OD 490 nm ± SD and statistical differences (*P* < 0.05) are indicated by*

Sera induced in *T. gondii* immunized mice were tested post-immunization by standard ELISA at weeks 0, 2, 4, and 6. As shown in Fig. 5, significantly higher levels of total IgG antibodies were detected in the sera of mice immunized in the experimental groups compared with the control immunized groups, especially after the third immunization. Moreover, the mean titers of IgG antibodies induced in the alum-MPLA- toxofilin group were significantly higher after both priming and booster immunization compared with the levels induced by toxofilin alone DNA vaccine or with single adjuvant. In contrast, the total IgG levels were similar between alum-toxofilin and MPLA-toxofilin groups.

**Fig. 6:**
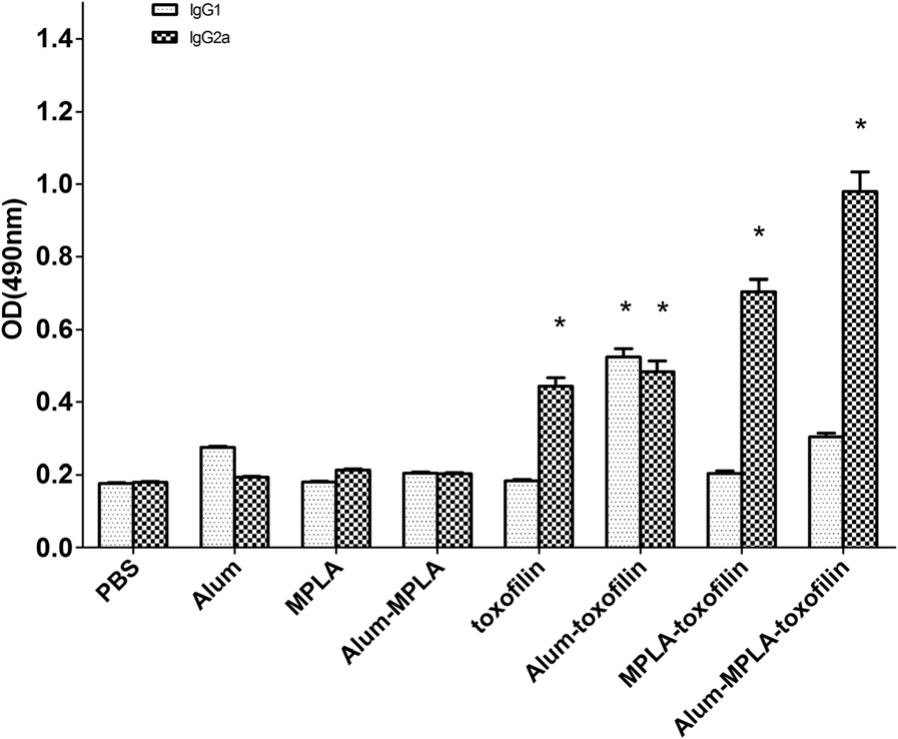
Distribution of IgG subtypes IgG1 and IgG2a in immunized mice. The levels of IgG subtypes IgG1 and IgG2a in sera of mice 2 weeks after the last immunization were analyzed by ELISA. Results are expressed as means of the OD 490 ± SD and statistically significant differences (*P* < 0.05) are indicated by an asterisk (*)

Serum levels of IgG1 and IgG2a, which are characteristic of Th2 and Th1 responses respectively, were analyzed by ELISA after the last immunization. As shown in Fig. 6, the non-adjuvanted toxofilin DNA vaccine group demonstrated similar levels of IgG1 and higher levels of IgG2a compared with control groups. However, compared with the non-adjuvanted toxofilin DNA vaccine, the alum-toxofilin group showed a significant improvement in the IgG1 level, indicative of a Th2-skewed immune response. Conversely, the MPLA-toxofilin group induced higher levels of IgG2a, and is therefore more likely to induce a Th1-biased immune response. Importantly, significantly higher serum levels of IgG2a and a slight increase in IgG1 were detected in the alum-MPLA-toxofilin immunized group when compared with other groups, suggesting that this combination of DNA vaccine and adjuvant may be most efficacious in raising a Th1 response following immunization.

### Cytokine production by spleen cells

The sera samples collected were used to measure the levels of IFN-γ, IL-4, and IL-10 induced by immunization with toxofilin DNA vaccine in the presence or absence of adjuvants. Generally, IFN-γ favors Th1-type immune responses, whereas IL-4 and IL-10 drive Th2 skewing [16]. As demonstrated in Table 3, values of IFN-γ in toxofilin DNA vaccine, alum- toxofilin, and MPLA- toxofilin immunization groups were 607.29 ± 63.09 pg/mL, 694.39 ± 88.64 pg/mL, and 3043.54 ± 350.72 pg/mL, respectively, which were significantly higher than in the control group of PBS (48.54 ± 2.40 pg/mL), alum (49.58 ± 2.37 pg/mL), MPLA (218.34 ± 21.34 pg/mL), alum-MPLA (267.34 ± 24.35 pg/mL). Furthermore, the highest level of IFN-γ was detected at 3859.42 ± 380.67 pg/mL in the alum-MPLA-toxofilin group. Similarly, elevated levels of IL-4 and IL-10 were detected in the alum-toxofilin, MPLA- toxofilin, and alum-MPLA-toxofilin groups. However, significantly higher concentrations of both cytokines (IL-4 236.473 ± 24.05 pg/mL, IL-10 212.32 ± 30.27 pg/mL) were detected in the alum-toxofilin group, compared with the other groups.

**Table 3:** Cytokine production by splenocyte^a^ cultures from immunized BALB/c mice

Groups	Cytokine Production (pg/ml)^b^		
	IFN-γ	IL-4	IL-10
PBS	48.54 ± 2.40	38.79 ± 2.28	44.70 ± 2.70
Alum	49.58 ± 2.37	110.49 ± 10.38	123.45 ± 13.48
MPLA	218.30 ± 21.34	40.35 ± 2.34	51.46 ± 3.21
alum-MPLA	267.34 ± 24.35	78.34 ± 7.34	58.46 ± 5.34
pEGFP- toxofilin	607.29 ± 63.09^*^	37.61 ± 2.87	34.70 ± 3.04
alum-toxofilin	694.39 ± 88.64^*^	236.47 ± 24.05^**^**^	212.32 ± 30.27^**^**^
MPLA-toxofilin	3043.54 ± 350.72^*#^	93.58 ± 10.31	85.43 ± 9.72
alum-MPLA-toxofilin	3859.42 ± 380.67^*#^	103.43 ± 24.43	115.32 ± 30.31

^a^Splenocytes from 3 mice per group two weeks after the final immunization

^b^Values for IFN-γ at 96 h, IL-4 at 24 h, IL-10 at 72 h and are expressed as mean ± SD

^*^Compared with PBS, alum, MPLA, aum-MPLA, *P* < 0.05

^#^Compared with alum-toxofilin or pEGFP-toxofilin groups, *P* < 0.05

^**^**^Compared with other groups

### T lymphocyte subsets analysis

The Splenocyte proliferation was evaluated in mice two weeks after the last immunization. As shown in Table 4, CD8+ T cells accounted for a significantly higher proportion than that in the control groups and a significant higher percentage of CD8+ T cells were dramatically increased in the alum-MPLA- toxofilin mixture group. The result also showed same statistically difference in the percentage of CD4+ T cells between experimental groups (toxofilin, alum-toxofilin, MPLA-toxofilin and alum -MPLA -toxofilin immunization groups) and the control groups.

**Table 4:** Percentages of T lymphocyte subsets in immunized mice^a^ by flow cytometry

Groups	CD3+ CD4+ CD8− (%)	CD3+ CD8+ CD4− (%)
PBS	15.31 ± 0.12	7.35 ± 0.18
alum	17.34 ± 0.34	9.62 ± 0.29
MPLA	17.92 ± 0.25	9.08 ± 0.24
alum-MPLA	18.49 ± 0.19	10.13 ± 0.30
pEGFP- toxofilin	20.17 ± 0.53^*^	12.48 ± 0.43^*^
alum-toxofilin	21.37 ± 0.76^*^	12.98 ± 0.45^*^
MPLA-toxofilin	27.46 ± 0.97^*#^	17.39 ± 0.98^*#^
alum-MPLA-toxofiln	32.38 ± 1.19^*#^	20.68 ± 1.37^*#^

^a^Splenocytes from 3 mice per group at the fourth week after the final immunization

^*^Compared with PBS, alum, MPLA, alum-MPLA. *P* <0.05

^#^Compared with alum-toxofilin or pEGFP-toxofilin groups, *P* <0.05

Results are presented as means ± SD of three replicate determinations

### Protective immunity analysis against challenge with T. gondii RH strain

**Fig. 7:**
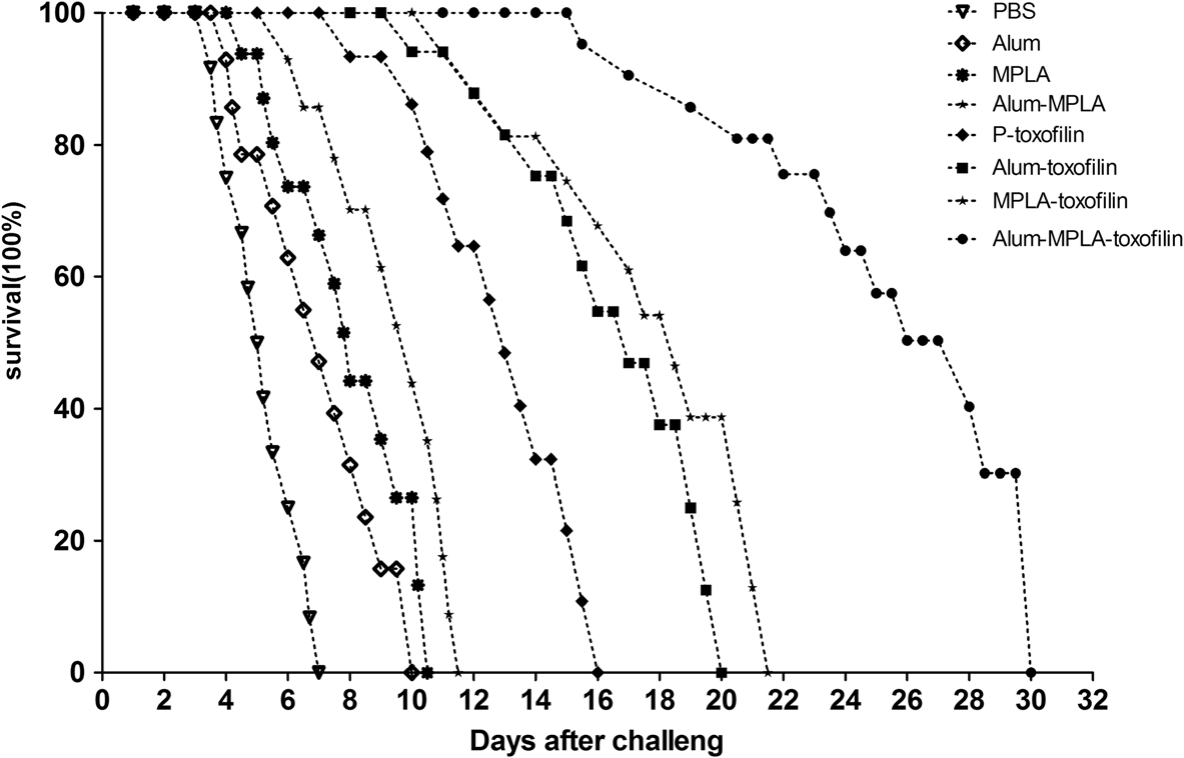
Survival curves of the vaccinated BALB/c following *Toxoplasma gondii* challenge infections. The mice in eight groups of mice were challenged with 1 × 10^4^ tachyzoites of the virulent RH strain of *T. gondii* 2 weeks after the last immunization (*n* = 12) per group

The survival curves against acute toxoplasmosis are shown in Fig. 7, and notably prolonged survival times were recorded in the experimentally immunized mice in comparison with the control groups (*P* < 0.05). Among the different experimental groups, mice vaccinated with the alum-MPLA-toxofilin had obviously prolonged survival, with 50% of mice surviving to day 24 of challenge.

“One month after challenge with 20 cysts of PRU strain of T. gondii, surviving mice were killed and brain cysts were enumerated.” The cyst number (1034.49 ± 45.87) in the mice immunized with alum-MPLA-toxofilin was significantly reduced compared to other groups (Table 5).

**Table 5:** Mean cyst burden per mouse brain 4 weeks after challenge with 20 cysts of *Toxoplasma gondii* PRU strain per mouse

Challenged group^a^	Brain cysts per mouse (mean ± SD)^b^
PBS	3042.43 ± 115.17
alum	2834.45 ± 100.89
MPLA	2767.36 ± 103.45
alum-MPLA	2532.56 ± 99.45
pEGFP-toxofilin	2048.79 ± 87.44^*^
alum-toxofilin	1764.17 ± 78.45^*^
MPLA-toxofilin	1563.42 ± 80.48^*^
alum-MPLA-toxofiln	1034.49 ± 45.87^*#^

^a^10 mice from each group were challenged intragastrically by 20 cysts 2 weeks after the last immunization. All samples were run for three times

^b^The mean number of cysts of each group was obtained from every mice brain cysts in the group

^*^Compared with PBS, alum, MPLA, alum-MPLA, *P* < 0.05

^#^Compared with pEGFP- toxofilin, alum-toxofilin and MPLA-toxofilin, *P* < 0.05

## Discussion

Bioinformatics has been regarded as crucial tool in the analysis of the protein characteristics including to the protein epitopes [27]. In this study, bioinformatic analysis has been applied to predict toxofilin antigens using relatedness software and online services. As shown in Fig. 1, the results of our analysis suggested that the toxofilin sequence is conserved between different strains of *T. gondii*. In addition, the 3D structure of toxofilin was constructed and several promising linear B- and Th-cell epitopes were identified. According to the data analysis, we concluded that toxofilin protein may be regarded as a potential antigen and we have successfully constructed an antigen-encoding plasmid, pEGFP-toxofilin. To further confirm the antigenicity of pEGFP-toxofilin after parasitic infection, the immune response (cellular and humoral) was assessed by immunization and lethal challenge.

Generally, infection with *T. gondii* induces a strong and persistent Th1 immune response. IFN-γ which is Th1-type cytokine has been regarded as a decisive mediator of resistance to *T. gondii* [28]. In addition, the principal source of IFN-γ is from both CD8+ T cells and CD4+ T cells [29]. In this study, the percentage of CD8+ T and CD4+ T cells were significantly increased, suggesting that the effect of synergy between CD8+ and CD4+ T cells may contribute to the higher concentration of IFN-γ. Previous studies have shown IFN-γ can also contribute to tryptophan degradation in a large number of infected cell types [29]. As shown in Table 3 and Table 4, by stimulation with the pEGFP-toxofilin DNA vaccine, a significant increase of IFN-γ, CD8+ T cells and CD4+ T cells, was induced in comparison with the control groups. Moreover, previous studies have suggested that the level of Th2-associated cytokines, IL-4 and IL-10, produced during *T. gondii* infection also play an important role in the immune response. In the present study, a slight reduction of both IL-4 and IL-10 was also observed in the pEGFP-toxofilin DNA vaccine group compared with the other groups, suggesting impaired Th2 responses compared with control groups.

Besides cellular immune responses, humoral immunity resulting in total IgG antibody plays an important role in controlling *T. gondii* infection [30]. In this report, we analyzed the humoral response after vaccination with pEGFP-toxofilin DNA vaccine and found that immunized mice presented high titers of total specific IgG in contrast to the control groups. Along with macrophages, specific IgG antibodies can protect the host from chronic *T. gondii* infections and cyst amount. Our observations were further validated by characterization of the mixed Th1/Th2 response. High IgG1 levels mainly indicate Th2 responses while elevated IgG2a levels favor development of Th1 responses. As shown in Fig. 6, we found higher levels of IgG2a and slightly lower IgG1 titers by the pEGFP-toxofilin DNA vaccine, indicating that our vaccine induced immune responses more biased towards Th1 in BALB/c mice, suggestive of protection against toxoplasmosis and therefore, represents a potential vaccine candidate.

To strengthen and boost the level of molecular vaccines immune responses, adjuvant is a powerful tool to enhance the immunogenicity of DNA vaccines [31]. We evaluated the immunogenicity and protective capacity of pEGFP-toxofilin DNA vaccine either alone, with the single adjuvants alum or MPLA, or combined alum-MPLA adjuvants. Incorporation of alone or mixed adjuvants significantly boosted the production of IgG titers following vaccination with the pEGFP-toxofilin DNA vaccine, inducing differential skewing of protective immunity compared with the non-adjuvanted toxofilin antigen, and to each adjuvant, as alum and MPLA induce different biases in the immune response [17, 18]. The alum-toxofilin group increased IgG1 levels compared with non-adjuvanted toxofilin, skewing the immune response towards a Th2 response associated with strong production of the IL-4, IL-10, and the IgG1 antibody subtype. Conversely, the MPLA adjuvant is known to induce a Th1-biased immune response [32], resulting in significantly increased specific serum IgG2a isotype antibodies and higher levels of Th1 cytokines. Importantly, the alum-MPLA-toxofilin could effectively lead to a shift from a Th2-skewed immune response towards a Th1 profile and, ultimately, to a balanced immune response compared with the use of a single adjuvant.

For protective efficacy of DNA vaccines against *T. gondii* challenge, the most direct parameter including to survival time, brain cysts load, brain cysts reduction rate, and protection rate should be considered [33]. The present study revealed that the alum-MPLA-toxofilin group could prolong the mice survive time as shown in Fig. 7, significantly reducing the brain cyst burden shown in Table 5. However, our DNA vaccine did not provide complete protection at later time points. In future, evaluating the efficacy of vaccines from different sources remains a new and promising strategy [34]. Further studies are needed to continue to develop DNA vaccines to improve the immunization efficacy against toxoplasmosis.

## Conclusions

In the present study, we use bioinformatic approaches to analyze the antigenicity of *T. gondii* toxofilin antigen. Therefore, toxofilin could be regared as a novel and strong DNA vaccine candidate against toxoplasmosis. At the same time, immunization with the alum-MPLA mixture as an adjuvant, in combination with the pEGFP-toxofilin DNA vaccine, can enhance cellular immunity and shift the immune response towards a Th1 profile. Furthermore, the alum-MPLA-toxofilin can also induce partial protective immunity in the murine model.

## Abbreviations

DMEM: Dulbecco’s modified Eagle’s medium
HA: Hemagglutinin
Ig: Immunoglobulin
IL: Interleukin
ip: Intraperitoneally
MHC: Major histocompatibility complex
MPLA: Monophosphoryl lipid A
PBST: PBS containing 0.05% Tween20
TE: Total *T. gondii* antigen extract
Tg-toxofilin: Toxofilin protein of *T. gondii*
Th: T helper
